# Physical integrity and survivorship of long-lasting insecticidal nets distributed to households of the same socio-cultural community in Benin, West Africa

**DOI:** 10.1186/s12936-020-3138-7

**Published:** 2020-02-04

**Authors:** Idelphonse B. Ahogni, Albert S. Salako, Bruno Akinro, Arthur Sovi, Virgile Gnanguenon, Roseric Azondekon, Jean F. Dagnon, Pamela Akogbeto, Filémon Tokponon, Martin C. Akogbeto

**Affiliations:** 1 Centre de Recherche Entomologique de Cotonou (CREC), Ministry of Health, Cotonou, Benin; 2 Faculté des Sciences et Techniques, University of Abomey-Calavi, Abomey-Calavi, Benin; 3 International Chair of Physics, Mathematics and Application, ICPMA, Dangbo, Benin; 4 Faculté d’Agronomie, Université de Parakou (UP), Parakou, Benin; 5 USAID PMI Vector Link Project, Abt Associates, Bujumbura, Burundi; 6 University of Wisconsin Milwaukee, Milwaukee, USA; 7 President’s Malaria Initiative, US Agency for International Development, Cotonou, Benin; 8 National Malaria Control Program, Cotonou, Benin

**Keywords:** LLINs, Survivorship, Physical integrity, Malaria, Benin

## Abstract

**Background:**

Long-lasting insecticidal nets (LLINs) are designed to survive and sustain their physical barrier for 3 years in household conditions. However, studies have shown that most of these nets are usually torn or no longer present in the households in less than 3 years. This study was initiated in Benin to compare the survivorship and physical integrity of seven types of LLINs in a same socio-geographic area.

**Methods:**

In August 2017, 1890 households were selected in 9 villages in the municipality of Zagnanado in central Benin. Each one of the selected households received one of the seven LLIN products: Aspirational^®^, DawaPlus^®^ 2.0, OlysetNet^®^, PermaNet^®^ 2.0, PermaNet^®^ 3.0, Royal Sentry^®^ and Yorkool^®^. Overall, 270 LLINs of each type were freely distributed in Zagnanado, at a rate of 30 LLINs per type per village. These bed nets have been monitored and evaluated every 6 months to identify the most resilient and preferred LLINs in the community. Net survivorship was assessed using the rate of net loss and physical condition.

**Results:**

The survivorship of all types of LLIN was estimated at 92% (95% CI 90.33–92.96) after 6 months and 70% (95% CI 67.25–71.81) after a year of use. At 12 months, all bed nets monitored were below the NetCalc model threshold of 92.8% for an LLIN with a lifespan of 3 years. Only 1.73% of all types of LLIN had a visible loss of integrity after 6 months with a median proportionate hole index (PHI) estimated at zero. The percentage significantly increased after 12 months with 10.41% of damaged nets (all types of LLINs). The median PHI for each brand of net was 23, 196, 141, 23, 23, 121 and 72, respectively for Aspirational^®^, DawaPlus^®^ 2.0, OlysetNet^®^, PermaNet^®^ 2.0, PermaNet^®^ 3.0, Royal Sentry^®^ and Yorkool^®^. A significant difference was noted between the PHI at 6 and 12 months (p < 0.0001). After 12 months, the DawaPlus^®^2.0, OlysetNet^®^ and Royal Sentry^®^ suffered significantly more damage compared to the others (p < 0.001).

**Conclusion:**

The results of this study showed that after a year of use, the survivorship of the 7 LLIN products in households was lower than expected. However, all the LLIN products successfully met WHO standards for physical integrity after 12 months of use. The monitoring continues. The next steps will help to identify the most sustainable LLINs.

## Background

Malaria prevention using long-lasting insecticidal nets (LLINs) has significantly increased in sub-Saharan Africa in recent years. In many countries, the proportion of people sleeping under LLINs is high and the World Health Organization (WHO) recommended universal coverage goal (one net for every two people) is on track [1]. The current challenge is to sustain this high coverage rate. Therefore, the survivorship and physical integrity of LLINs are two important indicators determining the regularity of the mass distribution campaigns of LLINs. For this reason, the WHO recommends that countries monitor LLINs following the mass distribution campaigns [1].

In 2013, the WHO provided technical recommendation to estimate the physical integrity of nets and determine the median lifespan of LLINs using various information such as the presence or absence of LLINs, in households, the rate of tearing of LLINs [2, 3]. Past studies have already followed this recommendation and measure the effectiveness of various brands of LLINs in different ecological and socio-cultural areas [4, 5]. The heterogeneity of the findings reported from those studies suggest that the survivorship and physical integrity of LLINs can vary considerably from 2 to 4 years [6], more than 4 years or even less [7]. The differences in findings are suspected to be influenced by cultural, environmental, behavioural factors and the brand of the LLIN [8–10]. These observations were confirmed by a study in Nigeria, where a public health behavioural change intervention has significantly improved household attitudes towards net maintenance and repair [11]. Likewise, in Mozambique, a study comparing the lifetime of two types of LLINs between 2008 and 2011 in Nampula found that 100-denier polyester LLINs were much more effective than 150-denier polyethylene LLINs [12]. This study also showed that nets were more effective in households far from the sea shores (located in the interior of the country) than those located along the coast.

Many donors including the Global Fund and the US President’s Malaria Initiative, are keen to encourage innovation and the production of LLINs. However, the production and marketing of new LLINs face several challenges. Currently, there are few specifications on existing LLINs, which do not allow manufacturers to develop new products. In addition, the LLIN procurement process does not motivate the production of better LLINs since procurement agencies are generally limited to purchasing cheaper nets that meet the minimum requirements of WHOPES. In this context, the evaluation of new prototype of nets under field conditions is necessary to determine their real lifespan.

In this study, the survivorship and physical integrity of 7 types of LLINs under field conditions were assessed. The recorded data were correlated with the characteristics (3-year efficacy in terms of survivorship and physical integrity according to the manufacturer) of each type of LLIN in order to highlight the types of nets that are more resistant to extrinsic factors.

## Methods

The study was conducted from August 2017 to September 2018 and aimed to compare the field performance of the LLINs and yield programmes with evidences needed to strengthen their procurement, delivery and effectiveness.

The study design is a prospective study of a cohort of seven types of LLIN. The types of LLIN included DawaPlus^®^ 2.0, OlysetNet^®^, PermaNet^®^ 2.0, PermaNet^®^ 3.0, Aspirational^®^, Royal Sentry^®^, and Yorkool^®^, which were randomly distributed, free-of-charge, in the selected compounds by door-to-door visit in a same socio-cultural community and followed every 6 months.

### Community counseling

Community level meetings were organized to educate all the people in the selected communities on the adverse consequences of malaria, the benefits of using long-lasting nets, correct handling and use of nets in line with WHO recommendation and the need for reporting any adverse events.

The study area was selected in consultation with the malaria control programme. Village level meetings were organized to obtain permission to use the community as a study site, to inform the community members of the study objectives and methodologies and benefits of the study, to seek community acceptance for use; and to seek their support in successful conduct of the study.

People were informed that nets to be provided have labels stitched and they have been marked with water-soluble ink. They were told why such marking has been made in the interest of transparency. They were asked not to remove the identification labels from the nets.

Informed consent was obtained from all heads of households enrolled in the study at the time of census survey when all potential households were visited by a team of investigators.

### Study area

**Fig. 1:**
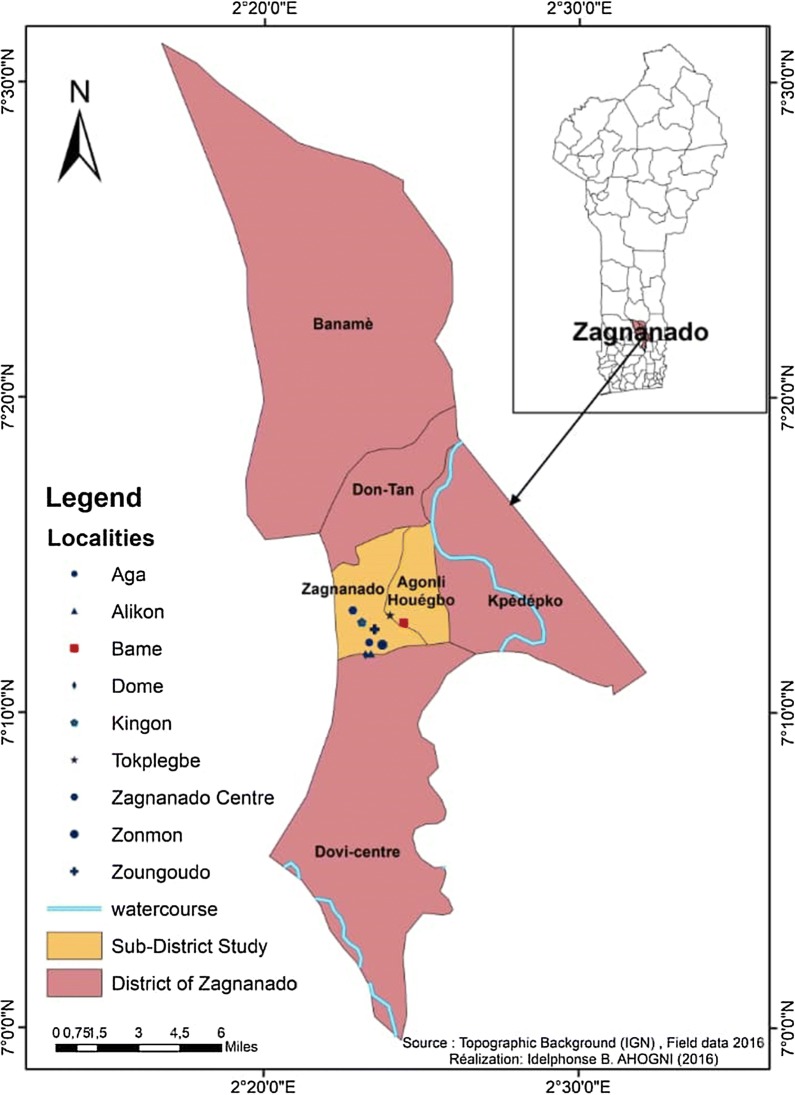
Map of Benin showing the 9 study villages

The study was conducted in Zagnanado, a municipality of the department of Zou in central Benin, about 180 miles from Cotonou, the economic capital of Benin. The average annual rainfall in this region is 1200 mm/year while the average daily temperature varies between 25 and 32 °C. The choice of Zagnanado is motivated by the fact that many villages are rice growing areas with a high density of *Anopheles* mosquitoes all year round. People living in Zagnanado are used to sleeping under mosquito bed nets every night to avoid mosquito bites. In this municipality, a total of 9 selected villages were surveyed as part of this study. Height of these villages (Aga, Alikon, Dome, Kingon, Tokplegbe, Centre Zagnanado, Zoungoudo and Zonmon) are located in the Zagnanado district and the last one (Bame), is located in the near the municipality of Agonli-Houegbo (Fig. 1). The ecological and socio-cultural characteristics of the selected villages are similar because they belong to the same geographical area. Most of the population of the 9 villages are of the same ethnic group. They speak the same language and engage in the same professional activities (agriculture, small commerce, handiwork).

### Inclusion/exclusion criteria

Villages in this area average approximately 200 compounds with approximately 2 sleeping spaces per compound. All houses are eligible to participate regardless of whether they currently own a WHO-recommended long-lasting insecticidal net, or not. Participants have retained any nets they currently own but have been asked to use the nets provided.

### Preparatory phase of the study before the distribution of the LLINs

Prior to the distribution of the seven types of LLINs, a household census study was conducted to generate a master list for household’s selection and random allocation of LLINs for follow-up. Six teams composed of two researchers and a chief of villages conducted the census of all the households. The census collected the information using WHO/LLINs durability guidelines questionnaire.

After the census, the household selection, the distribution of consent forms and informed briefing note on LLINs, the distribution operation was conducted. The operation aimed at generating a master list with a random possession of the LLINs by households for their follow up.

The information note is addressed to the occupants of the selected sleeping areas. The occupant is the person who sleeps at the sleeping area where the net will be installed or the representative of those who use the same sleeping area and the same net. The occupant is the user of the net. The information note covered: the objectives of the study, the steps in the study, information on the nets to be distributed, voluntary participation, monitoring of the nets, risks, adverse reactions and benefits of the study, the right to refuse or withdraw at any time from the study, confidentiality related to such a study.

### Distribution of the LLINs to households

Three teams composed of two researchers of the Center of Entomological Research of Cotonou (CREC) and a chief of the concerned village have freely distributed the LLINs to selected households during door-to-door visits. Households were allowed to keep their old bed nets, while being encouraged to use the newly distributed ones. Each household received only one LLIN and was free to withdraw from the study at any time. All LLINs distributed were labelled twice (first with a wash-resistant ink and second with water-soluble ink as a quality control for the approximate evaluation of the number of washes), and bore a unique identification number. During the distribution, the name of the village, the name of the head of the household, the identification number of the household, the identification number of the mosquito net were registered. The coordinates from the global positioning system (GPS) were also recorded to help identify household during follow-up. Names of the interviewer have also registered for the quality assurance.

Seven types of MILDs were distributed:

- DawaPlus^®^ 2.0, in 100% polyester, 75 ± 100 denier, with deltamethrin incorporated in the fibers (80 mg of active ingredient: ai/m^2^). The size of the meshes of the net is of 25 holes/cm^2^.
- OlysetNet^®^, in 100% polyethylene, 150 denier, with incorporated permethrin (50 mg ai/m^2^). The size of the meshes of the net is of 25 holes/cm^2^); OlysetNet^®^ is characterized by large meshes (4 mm × 4 mm) with 10 holes/cm^2^ as a minimum.
- PermaNet^®^ 2.0, in 100% polyester, the fibers of the net are 75 or 100 denier. The size of the meshes of the net is 25 holes/cm^2^ as a minimum, with incorporated deltamethrin (55 mg ai/m^2^).
- PermaNet^®^ 3.0, in 100% polyester (faces) and 100% polyethylene (roof), the fibers of the net are 75 or 100 denier. The mesh size of the net is at least 21 holes/cm^2^. PermaNet^®^ 3.0 is an LLIN with incorporated deltamethrin (66 mg ai/m^2^) (coated on filaments) and butoxide piperonyl (PBO) on the roof.
- Aspirational^®^, in 100% polyester, 150 denier, with incorporated alphacypermethrin (66 mg a.i/m^2^). The size of the meshes of the net is of 18 holes/cm^2^.).
- Royal Sentry^®^ in 100% polyethylene, the fibers of the net are 75 or 100 denier. The mesh size of the net is 130 holes/cm^2^ minimum, with incorporated alphacypermethrin (261 mg ai/m^2^).
- Yorkool^®^ LN, in 100% polyester, the fibers of the net are 75 or 100 denier. The mesh size of the net is 24 to 26 holes/cm^2^, covered with deltamethrin (55 mg ai/m^2^). The last three LLINs listed are experimental prototypes of LLINs.

### Sample size

A sample of 250 LNs per product will allow detection of a 9%-point difference in LLIN attrition rate if the best-performing product has an attrition rate of 10%. This sample size will also allow detection of an 11%-point difference in LLIN attrition rate if the best-performing product has an attrition rate of 20%. An eight percent buffer has been added to the required sample size to preventive any negative impact of a mid-course withdrawal of some study participants. So, a sample size of 270 LLIN per brand was retained for the study.

### Cohort study

Monitoring generally follows the guidelines from the WHO Pesticide Evaluation Scheme on the evaluation of LLINs (WHOPES 2011) with modifications as recommended by the durability of LLINs workstream of the Vector Control Working Group of Roll Back Malaria. Insecticidal activity has not been monitored, as the physical condition of the nets is considered the limiting factor in net longevity.

A cohort of approximately 270 nets of each type (one per household) has been followed in the enrolled villages. The same nets in the cohort was observed every 6 months. The purpose of the cohort study is to accurately assess net survivorship and declining fabric integrity while recognizing that the results may be biased due to participant knowledge that they are being observed (i.e. the Hawthorne effect). In combination with the cross-sectional surveys, this may more accurately estimate net loss and deterioration.

### Monitoring indicators

#### Survivorship of LLINs

Overall, the survival rate of the different types of LLINs was assessed by the following formula:

$$\frac{{{\text{Number}}\;{\text{of}}\;{\text{LLINs}}\;{\text{in}}\;{\text{good }}\;{\text{condition}}}}{{{\text{Total}}\;{\text{number}}\;{\text{of}}\;{\text{distributed}}\;{\text{LLINs}} - {\text{Total}}\;{\text{number}}\;{\text{of}}\;{\text{displaced}}\;{\text{or}}\;{\text{absent}}\;{\text{LLINs}}}} \times 100.$$

Households closed during an assessment visit are censored using the Kaplan–Meier non-parametric survival analysis method [13]. The survival of nets over time is compared to the 2- and 3-year life expectancy models developed by NetCalc (http://www.networksmalaria.org) and recommended by Roll Back Malaria. The equations used to calculate the loss rates associated with the absence of LLINs or displacement of LLINs from the households are as follows:

- Attrition-1: (physical damage)
  $$\frac{{{\text{Total}}\;{\text{number}}\;{\text{of}}\;{\text{LLINs}}\;{\text{no}}\;{\text{longer}}\;{\text{in}}\;{\text{use}}\;{\text{because}}\;{\text{of}}\;{\text{physical}}\;{\text{damage }}}}{{{\text{Total}}\;{\text{number}}\;{\text{of}}\;{\text{distributed}}\;{\text{LLINs}}}} \times 100.$$
- Attrition-2: (displacement)
  $$\frac{Total\;number\;of\;stolen,\; given,\;or\;sold\;LLINs}{Total\;number\;of\;distributed\;LLINs} \times 100.$$
- Attrition-3: (other attrition reasons)
  $$\frac{Total\;number\;of\;LLINs\;no\;longer\;in\;use\;due\;to\;other\;reasons}{Total\;number\;of\;distributed} \times 100.$$

#### Integrity of the net

The physical integrity of all nets in each village was assessed every 6 months for absence/presence of holes. The observed holes were categorized into 4 types:

- T1 size holes (smaller than one inch: 0.5–2 cm).
- T2 size holes (larger than an inch, but smaller than a fist: 2–10 cm).
- Holes of size T3 (larger than a fist but smaller than a head: 10–25 cm).
- T4 size holes: (larger than a head: > 25 cm).

The integrity of each type of LLIN was determined by two indicators:

- The proportion of nets with any hole:
  $$\frac{{{\text{Total}}\;{\text{number}}\;{\text{of}}\;{\text{LLINs}}\;{\text{with}}\;{\text{holes}}}}{{{\text{Total}}\;{\text{number}}\;{\text{of}}\;{\text{LLINs}}\;{\text{in}}\;{\text{all}}\;{\text{households}}}} \times 100.$$
- The proportionate hole index (PHI) according to WHO guidelines [1]. This index is measured according to the formula: PHI = 1 × #T1 + 23 × #T2 + 196 × #T3 + 576 × #T4 (#T = number of holes in the size). It estimates the approximate value of the area occupied by the holes on each type of LLIN.

Based on the PHI obtained, each net was classified into the following three categories (Kilian/Roll Back Malaria: Measurement of Net Durability in the Field: Current Recommended Methodology, presented in Lyon, February 2012):

- In good condition (PHI ≤ 64),
- To be repaired (PHI ≤ 642) or
- To be replaced (PHI > 642) [2.3].

To assess the influence of user factors or proximity to a watercourse on the physical integrity of LLINs, the Binomial Negative Regression [14] was used with the PHI parameter as the variable of interest. An analysis of the type III variance of the regression [15] made it possible to assess the influence of each factor on the PHI.

### Statistical analysis

The data were processed with R Core Team software version 3.5.1 (2018). Survival and loss rates and their confidence intervals are calculated with the binomial test [16]. Proportion comparisons are obtained using the Chi^2^ test of multiple proportion comparison or two proportion comparison. Poisson regression, combined with the Wald coefficient test and the maximum likelihood test was performed using the PHI as outcome variable to determine the influence of population habits on the physical integrity of LLINs. Finally, a survival model based on the results of the various assessments was applied to the data on losses observed for various reasons during the different site visits made during the follow-up period of the LLINs.

### Ethical consideration

The protocol of this study was evaluated and approved by the National Committee on Ethics in Health Research (Approval No. CNERS 024 of 30 September 2015). The persons involved, freely gave their consent after being informed on the goals of the investigation, the minimal risks involved, the benefits and their freedom to participate.

## Results

### Net survivorship/attrition

Overall, 1890 LLINs were distributed at the beginning of the study. Six months (T6) and 12 months (T12) after distribution, 1617 and 1134 LLINs were found and evaluated respectively (Table 1). After 6 months, no significant differences were observed between the survival rates of the different LLIN types which were 90% for DawaPlus^®^ 2.0 and PermaNet^®^ 2.0, 91% for PermaNet^®^ 3.0, 92% for Aspirational^®^, OlysetNet^®^ and Royal Sentry^®^ and 93% Yorkool^®^ (p = 0.924) and after 12 months, these rates decreased to 67%, 73%, 67%, 72%, 71%, 69% and 67%, respectively for Aspirational^®^, DawaPlus^®^ 2.0, OlysetNet^®^, PermaNet^®^2.0, PermaNet^®^ 3.0, Royal Sentry^®^ and Yorkool^®^ (p = 0.723). Overall, the survivorship (all LLIN types combined) decreased to 91% at 6 months and 69% at 12 months, respectively (Table 1). There is a significant difference between the average survivorships of the 6th month and the 12th month (p < 0.0001).

**Table 1:** LLIN survivorship by assessment community

	District of Zagnanado							Total
	Aspir	Dawa 2.0	OlysetNet^®^	PN 2.0	PN 3.0	Roy S	Yorkool^®^	
Baseline (T0)								
Households enrolled	270	270	270	270	270	270	270	1890
After 6 months (T6)								
Households eligible	270	270	270	270	270	270	270	1890
Households opened	254	249	255	249	256	254	255	1772
Coded LLINs found	230	230	232	225	226	237	237	1617
Good/serviceable LLINs	229	230	231	225	225	236	237	1615
LLINs lost	24	19	23	24	30	17	18	155
Removed LLINs	20	15	19	19	24	13	15	125
Survivorship (%)	92	90	92	90	91	92	93	91
CI % 95	87.45–94.73	85.87–93.55	87.96–95.07	85.19–93.12	87.25–94.64	87.78–94.87	89.07–95.76	89.98–92.66
After 12 months (T12)								
Households eligible	246	251	247	246	240	253	252	1735
Households opened	181	202	189	192	187	192	188	1331
Coded LLINs found	158	164	160	167	166	162	157	1134
Good/serviceable LLINs	156	161	154	165	165	161	156	1118
(LLINs lost)	23	38	29	25	21	30	31	197
Removed LLINs	18	35	22	22	14	24	21	156
Survivorship (%)	67	73	67	72	71	69	67	69
CI % 95	60.79–73.24	66.81–78.91	60.76–73.29	65.76–77.76	64.83–76.86	62.73–74.97	60.23–72.67	67.17–71.73

*Aspir* Aspirational^®^, *Dawa 2.0* DawaPlus^®^ 2.0, *PN 2.0* PermaNet^®^ 2.0, *PN 3.0* PermaNet^®^ 3.0, *Roy S* Royal Sentry^®^

**Fig. 2:**
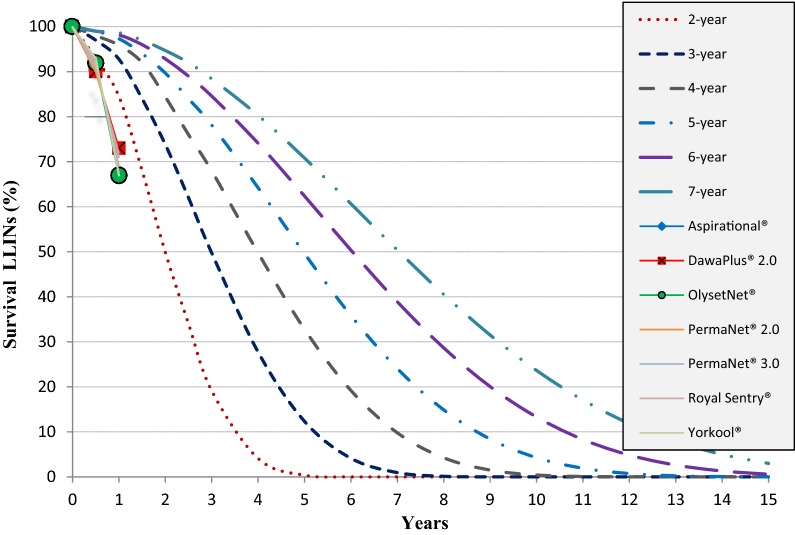
Estimated LLINs survival at 6 and 12, months in community compared with NetCalc model of curves of nets loss over 2, 3, 4,5, 6 and 7 years)

The observed survivorship was compared to that of the NetCalc model, which predicts a 92.8% survival of LLINs over a period of time. However, in this study, the 12-month survival rate (69%) is significantly lower than NetCalc’s predictions (Fig. 2).

### Reasons for loss of LLINs

A total of 155 LLINs were not found 6 months after distribution. After 12 months, the number of lost LLINs increased to 197. The main reason for the loss of bed nets was net displacement, which was mentioned125 times in the 6-month interviews and 156 times at 12 months (Table 2). Six months after distribution, no net was discarded due to physical damage. Less than 2% of the LLINs were physically deteriorated after 12 months of use. Less than 10% were moved from their original place in the same period. Only 2% were used for other purposes. There is no significant difference between the cumulative loss rates of the different types of nets (p = 0.938) after 12 months.

**Table 2:** Reasons for net loss (attrition): T_6_ and T12^a^ response summary

	District of Zagnanado							Total
	Aspir	Dawa 2.0	OlysetNet^®^	PN 2.0	PN 3.0	Roy S	Yorkool	
Households selected	270	270	270	270	270	270	270	1890
After 6 months (T6)								
(Lost LLINs)	(24)	(19)	(23)	(24)	(30)	(17)	(18)	(155)
Administered questionnaires	24	19	23	24	30	17	18	155
’’Physical damage’’ responses	0	0	0	0	0	0	0	0
’’Removal’’ responses	20	15	19	19	24	13	15	125
’’Re-purposed’’ responses	4	4	4	5	6	4	3	30
(%) Attrition rate-1	0	0	0	0	0	0	0	0
95% confidence interval	0	0	0	0	0	0	0	0
(%) Attrition rate-2	8.14	5.18	7.03	7.03	8.51	5.18	5.92	7.71
95% confidence interval	5.17–12.07	2.86–8.54	4.28–10.77	4.28–10.77	5.47–12.50	2.86–8.54	3.42–9.44	5.63–7.94
(%) Attrition rate-3	0.74	1.85	1.48	1.85	2.59	1.11	0.74	1.48
95% confidence interval	0.08–2.65	0.60–4.26	0.40–3.74	0.60–4.26	1.04–5.26	0.22–3.21	0.08–2.65	0.98–2.13
% of nets loss (total attrition)	8.88	7.03	8.51	8.88	11.11	6.29	6.66	8.2
95% confidence interval	5.77–12.93	4.28–10.77	5.47–12.50	5.77–12.93	7.62–15.48	3.71–9.88	3.99–10.33	7.00–9.53
After 12 months (T12)								
(Lost LLINs)	(23)	(38)	(29)	(25)	(21)	(30)	(31)	(197)
Administered questionnaires	23	38	29	25	21	30	31	197
’’Physical damage’’ responses	3	2	3	3	2	4	4	21
’’Removal’’ responses	18	35	22	22	14	24	21	156
’’Re-purposed’’ responses	2	1	4	0	5	2	6	20
(%) Attrition rate-1	1.11	0.74	1.11	1.11	0.74	1.48	1.48	1.11
95% confidence interval	0.23–3.21	0.09–2.65	0.23–3.21	0.23–3.21	0.09–2.65	0.41–3.75	0.41–3.75	0.69–1.69
(%) Attrition rate-2	6.67	12.96	8.15	8.15	5.19	8.89	7.78	8.25
95% confidence interval	4–10.33	9.20–17.56	5.18–12.08	5.18–12.08	2.86–8.55	5.78–12.94	4.88–11.64	7.05–9.59
(%) Attrition rate-3	0.74	0.37	1.48	0.00	1.85	0.74	2.22	1.06
95% confidence interval	0.09–2.65	0.01–2.05	0.41–3.75	0.00–1.26	0.60–4.27	0.09–2.65	0.82–4.77	0.65–1.63
% of nets loss (total attrition)	8.52	14.07	10.74	9.26	7.78	11.11	11.48	10.42
95% confidence interval	5.48–12.51	10.16–18.80	7.31–15.06	6.08–13.36	4.88–11.64	7.62–15.48	7.94–15.90	9.08–11.89

^a^Administered to all households if open and missing coded net

*Aspir* Aspirational^®^, *Dawa 2.0* DawaPlus^®^ 2.0, *PN 2.0* PermaNet^®^ 2.0, *PN 3.0* PermaNet^®^ 3.0, *Roy S* Royal Sentry^®^

### Physical integrity of LLINs

The average PHI of the different types of LLINs ranged from 1.05 to 6.49 and from 139.67 to 613.9, respectively after 6 and 12 months of use. Median PHI values for the 7 LLIN types ranged from 23 to 196 after 12 months of use. The classification of LLINs shows that more than 90% of the nets found were “in good condition” after 12 months regardless of the type. Overall, LLINs classified as “repairable” averaged less than 1% at 6 months and 3.44% at 12 months. Out of about 160 nets in each category seen in households, only 1 or 2 have reached a replacement stage. However, among OlysetNet^®^ nets, the number is higher (6/160). The same is true for DawaPlus^®^ 2.0 nets. The combined data from the 7 types of LLINs show that only 1.41% of these LLINs need to be replaced after 12 months of use (Table 3).

**Table 3:** LLIN fabric integrity (pHI) after 6 (T6) and 12 (T12) months at site

	District of Zagnanado							Total
	Aspir	Dawa 2.0	OlysetNet^®^	PN 2.0	PN 3.0	Royal S	Yorkool	
Households selected (T0)	270	270	270	270	270	270	270	1890
Tagged LLINs found (T6)	229	230	232	225	226	237	237	1616
n (%) of nets found with any hole (s)	2 (0.87)	5 (2.17)	5 (2.16)	5 (2.22)	7 (3.10)	4 (1.69)	2 (0.84)	30 (1.86)
CI 95 (%)	0.11–3.12	0.71–5.00	0.70–4.96	0.73–5.11	1.25–6.28	0.46–4.26	0.10–3.01	1.26–2.64
Mean pHI	1.05	3.95	6.49	0.75	5.46	5.43	3.67	23.13
Median pHI	0	0	0	0	0	0	0	0
IQR	0	0	0	0	0	0	0	0
n (%) of nets in pHI < 64 (‘good’ category)	228 (99.56)	226 (98.26)	229 (98.71)	224 (99.56)	223 (98.67)	235 (99.16)	235 (99.16)	1600 (99.01)
CI 95 (%)	97.59–99.99	95.61–99.52	96.27–99.73	97.55–99.99	96.17–99.73	96.99–99.90	96.99–99.90	98.40–99.43
n (%) of nets in pHI ≤ 642 (‘serviceable’ category)	1 (0.44)	4 (1.74)	2 (0.86)	1 (0.44)	2 (0.88)	1 (0.42)	2 (0.84)	13 (0.80)
CI 95 (%)	0.01–2.41	0.48–4.39	0.10–3.08	0.01–2.45	0.11–3.16	0.01–2.33	0.10–3.01	0.43–1.37
n (%) of nets in pHI > 642 ‘needs (‘replacement’ category)	0 (0)	0 (0)	1 (0.43)	0 (0)	1 (0.44)	1 (0.42)	0 (0)	3 (0.19)
CI 95 (%)	0.00–1.60	0.00–1.59	0.01–2.38	0.00–1.63	0.01–2.44	0.01–2.33	0.00–1.54	0.04–0.54
Tagged LLINs found (T12)	158	164	160	167	166	162	157	1134
n (%) of nets found with any hole (s)	10 (6.33)	20 (12.20)	29 (18.13)	17 (10.18)	12 (7.23)	20 (12.35)	10 (6.37)	118 (10.41)
CI 95 (%)	3.08–11.33	7.61–18.20	12.49–24.98	6.04–15.80	3.79–12.29	7.71–18.42	3.10–11.40	8.69–12.33
Mean pHI	566.3	314.1	613.9	193.9	139.67	329.25	235.8	370
Median pHI	23	196	141	23	23	121.1	72.5	46.5
IQR	17	205	589	174	39.75	570.25	405.5	411.5
n (%) of nets in pHI < 64 (‘good’ category)	156 (98.73)	152 (92.68)	144 (90.00)	160 (95.81)	163 (98.19)	152 (93.83)	152 (96.82)	1079 (95.15)
CI 95 (%)	95.50–99.85	87.57–96.16	84.27–94.18	91.55–98.30	94.81–99.63	88.94–97.00	92.72–98.96	93.73–96.33
n (%) of nets in pHI ≤ 642 (‘serviceable’ category)	0 (0)	9 (5.49)	10 (6.25)	5 (2.99)	2 (1.20)	9 (5.56)	4 (2.55)	39 (3.44)
CI 95 (%)	0.00–2.31	2.54–10.16	3.04–11.19	0.98–6.85	0.15–4.28	2.57–10.28	0.70–6.39	2.46–4.67
n (%) of nets in pHI > 642 ‘needs (‘replacement’ category)	2 (1.27)	3 (1.83)	6 (3.75)	2 (1.20)	1 (0.60)	1 (0.62)	1 (0.64)	16 (1.41)
CI 95 (%)	0.15–4.50	0.38–5.25	1.39–7.98	0.15–4.26	0.02–3.31	0.02–3.39	0.02–3.50	0.81–2.28

*Aspir* Aspirational^®^, *Dawa 2.0* DawaPlus^®^ 2.0, *PN 2.0* PermaNet^®^ 2.0, *PN 3.0* PermaNet^®^ 3.0, *Roy S* Royal Sentry^®^, *n* number, *LLINs* long-lasting insecticidal nets, *CI* confidence interval, *pHI* proportionate hole index

OlysetNet^®^ nets have a higher proportion of T1, T2 and T3 size holes compared to the other six types of LLINs (different letters: Table 4). On the other hand, the proportion of holes of size T1, T2 and T3 is similar for the other 6 types of LLINs. The 7 types of LLINs have similar proportions for T4 size holes (Table 4).

**Table 4:** Proportion of LLINs found with holes after 12 months

	Aspirational^®^	DawaPlus^®^ 2.0	OlysetNet^®^	PermaNet^®^ 2.0	PermaNet^®^ 3.0	Royal Sentry^®^	Yorkool^®^	p-value
(%) Proportion of holes of type 1	18.35a	15.85a	36.25b	14.37a	17.47a	18.52a	12.1a	< 0.001
(%) Proportion of holes of type 2	6.33a	11.59a	29.38b	9.58a	7.83a	5.56a	5.73a	< 0.001
(%) Proportion of holes of type 3	4.43ac	9.15a	23.75b	3.59ac	0.6c	1.85ac	3.18ac	< 0.001
(%) Proportion of holes of type 4	4.43a	3.05a	10a	1.8a	1.2a	6.17a	1.27a	< 0.0002

In each line, proportions with same letter are not statistically different

After classifying the LLINs by hole types, it appears that after 12 months of use, 7.96% had fabric tears, 0.68% had fabric tears due to burns and 0.23% had fabric tears in the seam and holes caused by rodents. OlysetNet^®^ and DawaPlus^®^ 2.0 nets are the two types of LLINs that had more tears in their fabric, respectively 15.34% and 9.41% (Table 5).

**Table 5:** Main causes of damage to LLINs at 6 and 12 months

	District of ZAGNANADO							Total
	Aspir	Dawa	OlysetNet^®^	PN2	PN3	Roy S	York	
After 6 months								
N (%) of nets found with any hole (s)	2 (0.86)	5 (2.17)	5 (2.16)	5 (2.22)	7 (3.09)	4 (1.69)	2 (0.84)	30 (1.86)
CI 95%	0.10–3.10	0.70–4.99	0.70–4.35	0.72–5.10	1.25–6.27	0.46–4.26	0.10–3.01	1.26–2.64
N (%) of nets with ‘rip in the fabric’	2 (0.86)	4 (1.73)	2 (0.86)	3 (1.33)	5 (2.21)	2 (0.84)	1 (0.42)	19 (1.17)
CI 95%	0.10–3.10	0.47–4.39	0.10–3.07	0.27–3.84	0.72–5.08	0.10–3.01	0.01–2.32	0.70–1.82
N (%) of nets with ‘burn holes’	0 (0)	1 (0.43)	1 (0.43)	1 (0.44)	1 (0.44)	1 (0.42)	1 (0.42)	6 (0.37)
CI 95%	0.00–1.59	0.01–2.39	0.01–2.37	0.01–2.45	0.01–2.44	0.01–2.32	0.01–2.32	0.13–0.80
N (%) of nets with ‘rip in the seam’	0 (0)	0 (0)	1 (0.43)	0 (0)	0 (0)	0 (0)	0 (0)	1 (0.06)
CI 95%	0.00–1.59	0.00–1.59	0.01–2.37	0.00–1.62	0.00–1.61	0.00–1.54	0.00–1.54	0.00–0.34
N (%) of nets chewing by rodent	0 (0)	0 (0)	0 (0)	1 (0.44)	0 (0)	0 (0)	0 (0)	1 (0.06)
CI 95%	0.00–1.59	0.00–1.59	0.00–1.57	0.01–2.45	0.00–1.61	0.00–1.54	0.00–1.54	0.00–0.34
After 12 months								
N (%) of nets found with any hole (s)	10 (5.5)	21 (10.4)	29 (15.3)	17 (8.9)	12 (6.4)	20 (10.4)	10 (5.3)	118 (10.41)
CI 95%	2.6–9.9	6.5–15.5	10.5–21.3	5.2–13.8	3.3–10.9	6.4–15.6	2.5–9.6	7.4–10.6
N (%) of nets with ‘rip in the fabric’	9 (4.97)	19 (9.41)	29 (15.34)	17 (8.85)	10 (5.35)	16 (8.33)	6 (3.19)	106 (7.96)
CI 95%	2.3–9.23	5.76–14.30	10.52–21.29	5.24–13.8	2.59–9.61	4.84–13.18	1.18–6.82	6.57–9.55
N (%) of nets with ‘burn holes’	1 (0.55)	0 (0)	0 (0)	1 (0.52)	1 (0.53)	3 (1.56)	3 (1.60)	9 (0.68)
CI 95%	0.01–3.04	0–1.81	0–1.93	0.01–2.87	0.01–2.94	0.32–4.5	0.33–4.59	0.31–1.28
N (%) of nets with ‘rip in the seam’	0 (0)	2 (0.99)	0 (0)	0 (0)	0 (0)	1 (0.52)	0 (0)	3 (0.23)
CI 95%	0–2.02	0.12–3.53	0–1.93	0–1.90	0–1.95	0.01–2.87	0–1.94	0.05–0.66
N (%) of nets chewing by rodent	0 (0)	1 (0.99)	0 (0)	0 (0)	1 (0)	1 (0.52)	1 (0)	4 (0.23)
CI 95%	0–2.02	0.12–3.53	0–1.93	0–1.90	0–1.95	0.01–2.87	0–1.94	0.05–0.66

*Aspir* Aspirational^®^, *Dawa 2.0* DawaPlus^®^ 2.0, *PN 2.0* PermaNet^®^ 2.0, *PN 3.0* PermaNet^®^ 3.0, *Roy S* Royal Sentry^®^, *N* Number, *CI* confidence interval

### Factors associated with loss of physical integrity

Table 6 provides household characteristics that may influence LLIN fabric wear and tear. These factors are related to the maintenance of LLINs, their users, the number of sleepers per net, the position of LLINs during the day, the location of the kitchen, the type of fuel used in the kitchen and the sleeping equipment (bamboo, bed, mat), and the presence of rodents. Overall, just over 64% of LLINs are well maintained regardless of the type of LLINs. About 60.49% of users were adults. In the majority of the households, LLINs were suspended (68.34%). More than 91.09% of households use wood for cooking and about 72.31% use it as sleeping material. A large majority of households (97.97%) acknowledged and reported the presence of rodents in their homes. Factors that show a significant relationship with the loss of physical integrity are: frequency of washing (high), type of cooking fuel (wood), LLIN user, number of sleepers per LLIN (n ≤ 5) and type of bedding (mat or bamboo) (p < 0.001) (Table 7).

**Table 6:** Percentage distribution of LLINs usage, housing characteristic by assessment visit (T6, T12)

	Aspir		Dawa 2.0		OlysetNet^®^		PN 2.0		PN 3.0		Roy S		Yorkool^®^		Total	
	T6	T12	T6	T12	T6	T12	T6	T12	T6	T12	T6	T12	T6	T12	T6	T12
LLINs maintenance																
Clean	63.32	63.92	68.70	65.85	63.79	64.38	64.89	65.27	64.16	68.07	71.31	60.49	67.09	65.61	66.21	64.81
Dirty	36.68	36.08	31.30	34.15	36.21	35.62	35.11	34.73	35.84	31.93	28.69	39.51	32.91	34.39	33.79	35.19
LLINs user																
Adult	65.07	59.67	60.87	69.80	54.31	60.85	57.78	61.98	57.52	58.82	59.92	67.71	60.76	60.64	59.47	60.49
Adult/children	32.75	37.02	36.96	30.20	41.81	34.92	40.89	33.85	41.15	37.43	37.13	29.17	38.40	36.70	38.43	36.33
Children	2.18	3.31	2.17	0.00	3.88	4.23	1.33	4.17	1.33	3.74	2.95	3.12	0.84	2.66	2.10	3.17
Number of sleeper/net																
0 < n ≤ 2	38.86	33.33	34.96	37.17	35.78	29.35	33.04	31.75	30.67	25.14	35.44	31.91	34.18	28.11	57.73	65.02
2 < n ≤ 5	38.43	44.25	43.81	39.27	45.26	52.72	45.09	47.09	48.00	52.46	42.62	44.68	47.26	48.65	34.63	34.10
5 < n ≤ 15	22.71	22.41	21.24	23.56	18.97	17.93	21.88	21.16	21.33	22.40	21.94	23.40	18.57	23.24	12.64	0.88
Daytime position of LLINs																
Hanging	79.48	65.82	82.61	71.95	79.74	68.75	78.22	68.86	85.84	71.08	79.75	67.90	80.59	63.69	80.88	68.34
Folded	12.23	25.95	13.04	22.56	12.50	26.25	11.11	25.15	8.41	23.49	13.92	24.69	11.81	27.39	11.88	25.04
Tidy away	8.30	8.23	4.35	5.49	7.76	5.00	10.67	5.99	5.75	5.42	6.33	7.41	7.59	8.92	7.24	6.61
Location of the kitchen																
Dehors	99.56	100.00	100.00	98.51	99.14	98.94	98.67	98.44	98.23	97.86	99.58	98.96	100.00	99.47	99.32	98.68
Interieur	0.44	0.00	0.00	1.49	0.86	1.06	1.33	1.56	1.77	2.14	0.42	1.04	0.00	0.53	0.68	1.32
Cooking fuel																
Firewood	86.90	92.27	91.30	90.10	90.95	89.42	91.56	91.15	89.82	88.24	88.19	92.19	89.45	92.55	89.73	91.09
Charcoal	9.61	7.73	6.52	8.91	6.03	10.05	4.89	8.33	6.64	11.76	8.86	7.29	6.75	5.85	7.05	8.38
Electricity	0.44	0.00	0.00	0.00	0.00	0.53	0.00	0.00	0.00	0.00	0.42	0.00	0.00	0.00	0.12	0.00
Gas	3.06	0.00	1.74	0.99	3.02	0.00	3.56	0.52	3.10	0.00	2.53	0.52	3.80	1.06	2.97	0.44
Other	0.00	1.10	0.00	3.96	0.00	2.65	0.00	2.60	0.00	1.60	0.00	2.08	0.42	2.66	0	0.00
Sleeping material																
Bambou	11.35	3.87	10.43	6.93	14.22	6.35	9.33	5.73	9.29	5.88	9.28	7.29	10.55	4.79	10.64	6
Bed	78.17	72.93	73.04	69.31	70.69	70.90	76.00	66.15	77.88	70.05	73.42	68.23	74.26	66.49	74.75	72.31
Matting	10.48	22.10	16.52	19.80	15.09	20.11	14.67	25.52	12.83	22.46	17.30	22.40	14.77	26.06	14.60	21.69
Presence of rodents																
No	2.62	1.85	2.17	2.26	3.88	4.12	3.11	1.14	2.65	2.33	3.38	2.86	2.11	1.18	2.85	2.03
Yes	97.38	98.15	97.83	97.74	96.12	95.88	96.89	98.86	97.35	97.67	96.62	97.14	97.89	98.82	97.15	97.97
Total number of households visited/opened	229	158	230	164	232	160	225	167	226	166	237	162	237	157	1772	1134

*Aspir* Aspirational^®^, *Dawa 2.0* DawaPlus^®^ 2.0, *PN 2.0* PermaNet^®^ 2.0, *PN 3.0* PermaNet^®^ 3.0, *Roy S* Royal Sentry^®^, *LLINs* long-lasting insecticidal nets

**Table 7:** Factors associated to loss of physical integrity

Factors	Modalities	Coefficients	Rate ratio	CI-95%(RR)	p (Wald. Test)	p (LR-test)
Washing frequency	None	4.64	–	–	–	< 0.001
	1 time	− 1.48	0.23	[0.22–0.23]	< 0.001	
	2–5 times	− 1.52	0.22	[0.21–0.22]	< 0.001	
	6–10 times	− 1.06	0.35	[0.33–0.37]	< 0.001	
Cooking fuel	Firewood	3.53	–	–	–	< 0.001
	Charcoal	− 0.59	0.55	[0.53–0.58]	< 0.001	
	Electricity	− 14.84	0	[0–3.31]	0.932	
	Gas	− 2.19	0.11	[0.07–0.17]	< 0.001	
Sleeping material	Bet	3.34	–	–	–	< 0.001
	Matting	0.22	1.25	[1.22–1.28]	< 0.001	
	Bamboo	1.15	3.17	[3.09–3.26]	< 0.001	
LLINs maintenance	Clean	2.79	–	–	–	< 0.001
	Dirty	1.58	4.48	[4.77–4.98]	< 0.001	
Presence of rodents	No	3.004	–	–	–	< 0.001
	Yes	0.66	1.93	[1.76–2.11]	< 0.001	
LLINs user	Adult	3.14	–	–	–	< 0.001
	Adult/children	0.75	2.11	[2.07–2.15]	< 0.001	
	Children	0.83	2.3	[2.2–2.41]	< 0.001	
Sleeper/net	0 < n ≤ 2	2.24	–	–	–	< 0.001
	2 < n ≤ 5	1.02	2.78	[2.68–2.88]	< 0.001	
	5 < n ≤ 15	2.2	9	[8.69–8.31]	< 0.001	

The modalities with same letter are not different significatively

## Discussion

Unlike previous studies conducted in Benin [7, 17], this study evaluates the survivorship and physical integrity of various types of LLINs at the operational level and in the same socio-cultural context. Indeed, in previous work carried out in Benin, the different types of nets are distributed in different socio-cultural environments without taking into account the effect of the environment when comparing their performance [7, 17].

More than 70% of the seven types of LLINs survived after 12 months of use. This result is similar to that obtained by Azondekon et al. [12] and Gnanguenon et al. [7]. In another study conducted in Zambia [18], 90.4% of LLINs (PermaNet 2.0 and OlysetNet^®^) had also survived, 12 months post distribution. The same findings was reported by a study conducted in Nigeria (98.2% survival rate) [19] and another one in Rwanda (92% survival rate) [8] for polyester and polyethylene LLINs, 12 months post-distribution. When considering the physical integrity results of the LLIN tissue, the survivorship at 12 months was lower than that predicted by the NetCalc model (70% versus 92.8%). The 70% rate observed in this study could have been higher had “unrecovered” nets been taken into account. These nets may have been present in other houses, which were not visited. The further deterioration of OlysetNet^®^ noted in this study confirms the observations of Azondekon et al. [12] on polyethylene nets, which tend to suffer substantial physical damage. The same observation is made with Royal Sentry LLINs, which are also made of polyethylene. The search for the determinants of physical damage observed on LLINs revealed a number of associated factors such as: the number of sleepers often greater than 2 in a 2-place bed nets, the high frequency of washing (2 to 5 times in 6 months), the proximity of the kitchen to the beds, the type of sleeping equipment, and the poor maintenance of the nets. In addition, poor housing conditions and food conservation attract rodents that damage LLINs [21]. Similar results have been reported in Burkina Faso [20, 21].

Out of the 1134 LLINs surveyed during the visit after 12 months, 95.15% were “in good condition”, 3.44% “to be repaired” and 1.41% “to be replaced”. The proportion of T1, T2 and T3 holes is high among OlysetNet^®^ (polyethylene) nets, 36%, 29% and 23% respectively, only 12 months after distribution. A study conducted in Uganda, found that 33.7% of polyester nets had holes, 12 months post-distribution [5]. In Zambia, 9.6% of polyester and polyethylene nets were classified as “To be replaced” after 12 months under field conditions [18], which is a relatively higher proportion compared to the observations reported here where only 1.41% of polyester and polyethylene LLINs were widely torn and required replacement. It has been shown that LLINs with holes (64 < PHI < 642) can prevent mosquito bites due to the repellent effect of pyrethroids [5]. However, it is possible that nets may become ineffective when the area occupied by the holes reaches a certain threshold [12].

In this study, after 12 months of use, apart from 6 OlysetNet^®^ to be replaced out of 270 distributed OlysetNet^®^, all other nets were in good condition. The relatively high loss rate noted only after 12 months of net use may be related to the low standard of living of the populations who were sometimes forced to move their nets to their farms or during travel to protect themselves from mosquito bites.

This study is unique in that it assesses the sustainability of 7 different types of LLINs in the same ecological and socio-cultural context, which is not the case with the same type of studies previously conducted in Benin. Displacement was the most frequent reason reported by the populations to justify the fact that a significant number of LLINs were unfound. The results of this study cannot be extrapolated to other brands of LLINs, even in similar contexts. In addition, further research is needed to determine the extent to which the survival and physical integrity of a LLIN brand affects its ability to prevent and reduce malaria transmission. It is also necessary to monitor new/experimental prototypes of LLINs in order to ensure that people adhere to their use. This monitoring makes it possible to evaluate the operational performance of these vector control tools and to ensure their quality compared to what is stated by the manufacturer. This can help the various decision-makers and partners involved in the fight against malaria to better guide the strategies to be implemented.

## Conclusion

The results of this study revealed that after 12 months of use the survivorship of the 7 types of LLINs in households was lower than expected. This low survivorship was mainly due to LLINs removal from households and was not related to the quality of the fabric. However, all the LLIN products successfully met WHO standards for physical integrity after 12 months of use. The authors suggest that the awareness campaigns on good practices for better LLINs use during each follow-up visit have contributed to the sustainability of the LLINs even after a year of usage. The monitoring continues. The next steps will allow us to identify the most sustainable LLINs.

## Data Availability

The data used and/or analysed in this study are available from the corresponding author on reasonable request.

## Abbreviations

CI: Confidence interval
CREC: Centre of Entomological Research of Cotonou
GPS: Global positioning system
ICPMA: International Chair of Physics, Mathematics and Application
LLIN: Long-lasting insecticidal net
NMCP: National Malaria Control Programme
PHI: Proportionate hole index
PMI: President’s Malaria Initiative
UP: Université de Parakou
USA: United States of America
USAID: United States Agency for International Development
WHO: World Health Organization
WHOPES: World Health Organization Pesticide Evaluation Scheme
